# Accurate, rapid and high-throughput detection of strain-specific polymorphisms in *Bacillus anthracis *and *Yersinia pestis *by next-generation sequencing

**DOI:** 10.1186/2041-2223-1-5

**Published:** 2010-09-01

**Authors:** Craig A Cummings, Christina A Bormann Chung, Rixun Fang, Melissa Barker, Pius Brzoska, Phillip C Williamson, Jodi Beaudry, Molly Matthews, James Schupp, David M Wagner, Dawn Birdsell, Amy J Vogler, Manohar R Furtado, Paul Keim, Bruce Budowle

**Affiliations:** 1 Life Technologies Corporation, Foster City, California, USA; 2 University of North Texas Health Science Center, Fort Worth, Texas, USA; 3 Northern Arizona University, Flagstaff, Arizona, USA; 4 Translational Genomics Research Institute, Phoenix, Arizona, USA

## Abstract

**Background:**

In the event of biocrimes or infectious disease outbreaks, high-resolution genetic characterization for identifying the agent and attributing it to a specific source can be crucial for an effective response. Until recently, in-depth genetic characterization required expensive and time-consuming Sanger sequencing of a few strains, followed by genotyping of a small number of marker loci in a panel of isolates at or by gel-based approaches such as pulsed field gel electrophoresis, which by necessity ignores most of the genome. Next-generation, massively parallel sequencing (MPS) technology (specifically the Applied Biosystems sequencing by oligonucleotide ligation and detection (SOLiD™) system) is a powerful investigative tool for rapid, cost-effective and parallel microbial whole-genome characterization.

**Results:**

To demonstrate the utility of MPS for whole-genome typing of monomorphic pathogens, four *Bacillus anthracis *and four *Yersinia pestis *strains were sequenced in parallel. Reads were aligned to complete reference genomes, and genomic variations were identified. Resequencing of the *B. anthracis *Ames ancestor strain detected no false-positive single-nucleotide polymorphisms (SNPs), and mapping of reads to the Sterne strain correctly identified 98% of the 133 SNPs that are not clustered or associated with repeats. Three geographically distinct *B. anthracis *strains from the A branch lineage were found to have between 352 and 471 SNPs each, relative to the Ames genome, and one strain harbored a genomic amplification. Sequencing of four *Y. pestis *strains from the Orientalis lineage identified between 20 and 54 SNPs per strain relative to the CO92 genome, with the single Bolivian isolate having approximately twice as many SNPs as the three more closely related North American strains. Coverage plotting also revealed a common deletion in two strains and an amplification in the Bolivian strain that appear to be due to insertion element-mediated recombination events. Most private SNPs (that is, a, variant found in only one strain in this set) selected for validation by Sanger sequencing were confirmed, although rare false-positive SNPs were associated with variable nucleotide tandem repeats.

**Conclusions:**

The high-throughput, multiplexing capability, and accuracy of this system make it suitable for rapid whole-genome typing of microbial pathogens during a forensic or epidemiological investigation. By interrogating nearly every base of the genome, rare polymorphisms can be reliably discovered, thus facilitating high-resolution strain tracking and strengthening forensic attribution.

## Background

Microbial forensics is a discipline with an epidemiological foundation focused on the characterization, analysis and interpretation of evidence derived from a disease outbreak to identify it as a criminal event (a biocrime, an inadvertent release or a hoax) and distinguish it from a natural disease outbreak (reviewed by Budowle *et al.*[1], Cummings and Relman [2]). Microbial forensic investigations seek to obtain information regarding the identification or source of the evidentiary material with the ultimate goals of identifying those responsible for the crime (that is, attribution), excluding innocent or unlikely sources, and reconstructing the events of a case. In this sense, a search for commonalities and clustering to identify the source of infection is similar to the standard epidemiologic methods used to investigate outbreaks of infectious diseases. Microbial forensic findings can be supportive for criminal prosecution or for actions that might be taken as a result of national policy decisions.

As an example of the use of genetic evidence, we briefly describe the aspects of the anthrax letter attack of 2001 (for further details see Amerithrax Investigation documents [3]). Emergence of several anthrax cases and the discovery of letters carrying *Bacillus anthracis *spores in the offices of several media outlets and the US Senate created an immediate need to obtain forensic evidence that could assist in identifying the source of the *B. anthracis *and ultimately the perpetrator(s) of this act. Genetic evidence was pursued to eliminate potential candidate sources of the *B. anthracis *spores, but identifying informative individualizing genetic markers was and is a challenge. At the time of the bioterrorist event, Sanger shotgun sequencing was the best approach for identification of genetic markers for attribution purposes. Because Sanger shotgun sequencing is costly and labor-intensive, only the genomes of a few (at most) selected reference samples and the evidence sample were sequenced [4-6]. Comparative genomics methods were then used to search for polymorphisms that could distinguish the samples to some degree, with the intention of designing targeted assays (for example, PCR or TaqMan^® ^assays) with which to screen the repository of collected candidate laboratory samples. Unfortunately, no such polymorphisms were initially discovered by this approach.

Culturing of the anthrax evidence eventually revealed that several variants with different colony morphologies were present as a mixture [3]. When these phenotypic variants were purified and characterized by shotgun sequencing, each was found to harbor a unique genetic variation. Real-time PCR assays targeting four variants in the anthrax evidence (designated A1, A3, D and E) were designed and used to screen the sample repository. Samples found to contain the same array of variants were considered to be more closely related to the evidence.

The strategy undertaken in response to the 2001 anthrax attacks suffered from several technical shortcomings. Results could not be obtained expeditiously, making impractical a rapid response during exigent circumstances, during which the threat of continued attack persisted [7]. Because of cloning and sequencing artifacts, and limited depth of coverage, many initial putative polymorphisms had to be verified by PCR and direct sequencing, which required additional time, cost and labor, and eliminated most of the initially identified 'polymorphisms'. Finally, by screening the approximately 1,000-sample *B. anthracis *investigation repository for a limited set of polymorphisms, which were identified by sequencing of a few selected isolates, most of the genome for most strains was simply not assayed, thus potentially missing information that might uniquely identify the source.

MPS technologies introduced in the past few years enable the generation of over 50 GB (and counting) of sequence data from a single instrument run, an example being the sequencing by oligonucleotide ligation and detection (SOLiD™) system from Applied Biosystems. This level of throughput makes feasible the sequencing of multiple bacterial genomes in a short period of time (approximately 1 week) and at a relatively low cost (< US$10,000 per sequencing run and much less on a per bacterial genome basis; reviewed in [8,9]). Before the introduction of this technology, time and cost constraints limited whole-genome sequencing to a small number of strains or isolates to identify potentially informative genetic markers. Characterization of additional strains, because of limited throughput and prohibitive costs, required screening a small selection of the polymorphic loci discovered by the initial comparison of the few complete reference genomes. Although this was the only viable approach for screening genetic variation between strains and isolates, there were two major limitations. The selected genetic marker sites may not be informative for the investigative question(s) and most of the genomes of the samples were underinterrogated, thus any other individualizing markers that may reside in the genome would go undetected. It is now feasible to sequence the entire genome of any culturable strain of interest rapidly and accurately for a reasonable cost using the current technology. The greatest level of resolution for strains is achieved by interrogating every base of the genome rather than restricting the search to selected loci.

When a closely related reference genome is available, an accurate consensus sequence can be derived by mapping fragment or mate-paired reads to a reference [9]. Referenced assembly has the advantage of leveraging the property of two-base encoding in SOLiD™ system data. Interrogating each base twice and requiring two adjacent colorspace differences significantly reduces error rates in detection of single-nucleotide polymorphisms (SNPs). In addition, the depth of coverage yields a greater accuracy for variant identification. We applied these methods to two bacterial species, *B. anthracis *and *Y. pestis*, to determine the possibility of sensitive and accurate detection of SNPs in closely related microbial genomes, analyzed in parallel.

## Results

### Validation of library barcoding

To facilitate the analysis of several strains on a single flow cell, genomes were indexed by ligating a unique six-base barcode to each library. The barcodes were sequenced during the SOLiD™ system run, then for each fragment read, the corresponding strain was assigned by matching to the known barcode sequences. Single mismatches in the barcode sequence were tolerated. Fidelity of the barcoding process was estimated after read mapping by identifying 27 unique SNPs in one *Y. pestis *strain with at least 25× coverage and then counting the number of reads overlapping the SNP that perfectly matched the reference genome or the other three *Y. pestis *genomes sequenced in this run. At these 27 positions, 2,274 reads matched the consensus (that is, the non-reference allele), whereas only 6 reads matched the reference allele, suggesting that they could be derived from one of the other three *Y. pestis *genomes in the experiment. Normalized for library representation, the maximum estimated rate of 'bleed-through' between libraries was 0.076%.

### *B. anthracis *resequencing

**Figure 1:**
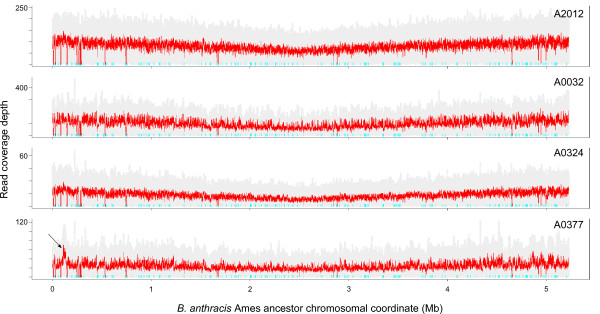
**Unique coverage plots of *B. anthracis *genomes**. Reads were mapped to the *B. anthracis *Ames ancestor chromosome using mapreads. Each row represents a *B. anthracis *chromosome (strain indicated at the upper right of each plot). Grey dots indicate coverage values at each position and red lines indicate the running mean with a window size of 1,000. Cyan tick marks near the × axis indicate the location of non-unique 35-mers in the chromosome: no reads can map uniquely to these sequences. The arrow in the A0377 plot indicates the amplified genomic region depicted in detail in Figure 4. The V-shaped profile of the coverage plot is due to the fact that replicating bacteria initiate a second round of chromosomal replication before the completion of the ongoing round, resulting in higher copy number at the origin of replication (at the ends of the plot) and lower copy number at the terminus (center of the plot).

Four *B. anthracis *strains were chosen for sequencing on the SOLiD™ system: A2012, a culture of the Ames ancestor strain from which the published genome sequence is available, and three related, previously unsequenced strains from the branch A lineage (A0032, A0324 and A0377) [10-12]. For each strain, fragment libraries were constructed. The four *B. anthracis *libraries were run on a single flow cell together with four *Y. pestis *libraries (see below), such that each library occupied approximately one-eighth of a flow cell. Between 4 million and 26 million reads of 35 bp were obtained for each *B. anthracis *strain, with the difference in number of reads most likely being due to inaccurate quantification of genomic DNA or individual library preparations. When aligned to the Ames ancestor reference chromosome, median read depth ranged from 16 for A0324 to 93 for A0032 (Table 1, Figure 1). Median coverage of the two virulence plasmids suggested mean copy numbers of 1.8 to 3.1 for pXO1 and 1.5 to 1.8 for pXO2 per chromosome. Fewer than 100,000 reference bases were not covered by a uniquely matching read, with most of these bases being found in repetitive or multi-copy genomic sequence (for example, rRNA loci).

**Table 1:** *B. anthracis *SOLiD™ system sequencing statistics

	A0032 (China)	A0324 (Slovakia)	A0377 (Haiti)	A2012 (Florida Ames)
Beads found, n	25,789,050	3,849,205	5,393,062	19,073,351

Uniquely placed beads (≤ 3 mismatches), n (%)	15,561,852 (60.34)	2,803,450 (72.83)	4,020,634 (74.55)	13,447,970 (70.51)

Bases not uniquely covered, n (%)	74,268 (1.35)	95,241 (1.73)	78,849 (1.43)	68,629 (1.25)

Chromosome median coverage^a^	93×	16×	23×	79×

pXO1 median coverage	165×	34×	66×	241×

pXO1 mean copy number, n	1.8	2.1	2.9	3.1

pXO2 median coverage	135×	26×	42×	124×

pXO2 mean copy number	1.5	1.6	1.8	1.6

^a^2.0% of the reference genome is non-unique 35-mers.

×, times.

Alignment of reads to the Ames ancestor genome facilitated the detection of SNPs in each of the four *B. anthracis *strains (Table 2. See Additional File 1). Only a single nucleotide ambiguity, but no clear SNP, was identified for sample A2012, which has been verified as 100% identical to the Ames ancestor [4], yielding a false-positive rate of approximately 1 in 5 million (without sampling error correction), if the ambiguity is considered to be a SNP call.

**Table 2:** SNPs in *B. anthracis *strains, relative to Ames ancestor

	**A0032**	**A0324**	**A0377**	**A2012**
Chromosome				
SNPs, n	324	331	434	0
Ambiguities, n	11^a^	7^a^	13^a^	1
Total calls, n	335	338	447	1

pXO1				
SNPs, n	18	23	26	0
Ambiguities, n	0	1	0	0
Total calls, n	18	24	26	0

pXO2				
SNPs, n	10	8	11	0
Ambiguities, n	0	0	0	0
Total calls, n	10	8	11	0

^a^Four shared ambiguities in imperfect repeat region.

SNP, single-nucleotide polymorphism.

The other three *B. anthracis *strains each had between 352 and 471 unambiguous SNPs on the chromosome and two plasmids, with no more than 13 ambiguities, 4 of which were the result of misalignment of reads from an imperfect repeat sequence. As expected, the number of SNPs distinguishing any two strains was proportional to previous variable number tandem repeat (VNTR) predictions of evolutionary distance [10,13], with the most divergent strain (A0377) having the greatest number of variations. Ten private SNPs (that is, a, variant in only one strain in this set) were arbitrarily selected from each of the three non-Ames ancestor strains for validation by PCR amplification and Sanger sequencing. Because these SNPs were found in only one strain, they had a higher probability of being due to sequencing error than did SNPs common to more than one strain. All 30 of these SNPs were verified as true polymorphisms (data not shown).

**Figure 2:**
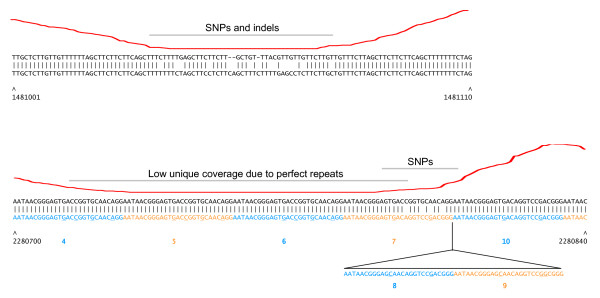
**Details of *B. anthracis *polymorphisms not detected by the SOLiD™ system**. Sequence alignments of *B. anthracis *Sterne (top line) versus Ames ancestor (bottom line). Red line indicates coverage depth of uniquely mapping A2012 SOLiD™ system reads at each position. Sterne chromosomal coordinates are given below the alignment. (Top) A region of high SNP density (13 SNPs in 40 bp) and small indels in GBAA_2450. (Bottom) In GBAA_1572, SNPs occur in a region containing a 27 bp repeat (alternating blue and orange blocks, with repeat number below). Relative to Ames ancestor, the Sterne genome lacks two repeat units. The four SNPs are adjacent to the indel breakpoint. Low unique coverage to the left of the SNPs is due to three perfect repeats (numbers 4, 5 and 6). Bases that differ from the consensus sequence are underlined.

To further assess false-negative and false-positive rates, the A2012 (the Ames ancestor) reads were aligned to the *B. anthracis *Sterne complete genome sequence. Results of SNP calling were compared with a "gold-standard" set of 150 SNPs, derived from comparative analysis of the two complete genomes as previously determined by Sanger sequencing [12]. The SNP detection software is able to identify heterozygous alleles, which is appropriate for diploid organisms, but because heterozygous alleles are not present in haploid organisms, heterozygous SNPs (ambiguous base calls) were not considered to be SNP calls for the purpose of this analysis. The SOLiD™ system analysis correctly identified 130 of the 150 SNPs (Table 3). Of the 20 SNPs that were not called, 17 were localized to two clusters that precluded alignment of reads to the reference. Thirteen of the false-negative SNPs are found in a stretch of 40 bp in the GBAA_2450 gene (Figure 2, top). Because only three colorspace mismatches were allowed in the read alignment step, this density of SNPs effectively precludes read mapping, so it is not possible to call SNPs. This region also contains small gaps that further prohibit matching of reads. The other cluster of false-negative SNPs (four SNPs in a 10 bp stretch) occurred in a 27 bp near-perfect direct repeat in the GBAA_1572 gene, encoding a penicillin-binding protein (Figure 2, bottom). Low unique-read mapping coverage in this region is due to the high density of SNPs and to the three perfect repeat copies to the upstream of the SNPs. The SNPs are also adjacent to a two-repeat unit insertion in the Ames ancestor genome relative to Sterne.

**Table 3:** SNP detection performance in *B. anthracis *A2012 versus Sterne

	FN^b^, n	FP^c^, n	Sensitivity, %	Specificity, %
Total	20	8	86.67	99.99985

Excluding SNP clusters^a^	3	8	97.74	99.99985

^a^17 SNPs in two clusters: 13/40 bp (GBAA_2450) and 4/10 bp (PBP).

^b^False negative.

^c^False positive.

SNP, single-nucleotide polymorphism.

**Figure 3:**
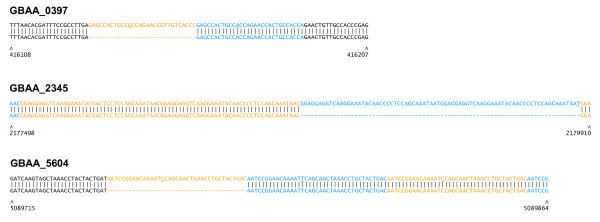
**Details of selected *B. anthracis *Sterne false-positive SNP calls**. Sequence alignments of (top) *B. anthracis *Sterne versus (bottom) Ames ancestor. Sterne chromosomal coordinates are given below the alignment. Repeat units are colored in alternating blue and orange text. Bases that differ from the repeat consensus sequence are underlined.

Eight putative false-positive SNPs (that is, not found in the gold-standard set) were called. Four of these were attributable to deletion of one or more direct repeat units from the Ames ancestor genome relative to Sterne [Ames ancestor open reading frame (ORF) IDs GBAA_0397, GBAA_2345, GBAA_4978, GBAA_5604] (Figure 3). Another false-positive SNP was in a transposase ORF (GBAA_5455) that is present in three nearly identical copies in each genome.

**Figure 4:**
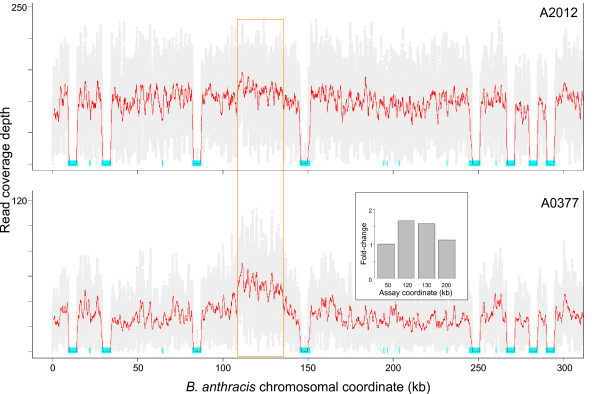
**Validation of a strain-specific copy number variant in *B. anthracis *A0377**. Unique coverage near the left end of (top) the A2012 and (bottom) A0377 chromosomes, is depicted as described in Figure 1. The orange box indicates the approximate boundaries of the amplified region in A0377. The eight large cyan bars correspond to the first eight rRNA loci in the genome, which are nearly identical to each other. TaqMan^® ^assays were designed, two internal to the amplified region and one to each side, with the approximate coordinates indicated on the X axis of the inset (in kb). These assays were run in triplicate on genomic DNA from each of the two strains, and relative fold-change of A0377 versus A2012 was calculated and normalized to the assay at coordinate 50 kb (inset).

In addition to SNP variants, coverage depth plots indicated a putative amplification on the A0377 chromosome in the region between the *rrnC *and *rrnD *loci (Figure 4). Two TaqMan^® ^assays inside the putative amplification and two flanking the region were designed. Quantitative real-time PCR assays on genomic DNA from A0377 vs. A2012 (Ames ancestor) indicated that the region was present at a copy number 1.6-fold to 1.7-fold higher than the rest of the chromosome, suggesting that it is present in multiple copies, at least in a fraction of the culture from which the genomic DNA preparation was obtained (Figure 4, inset).

### *Y. pestis *resequencing

**Figure 5:**
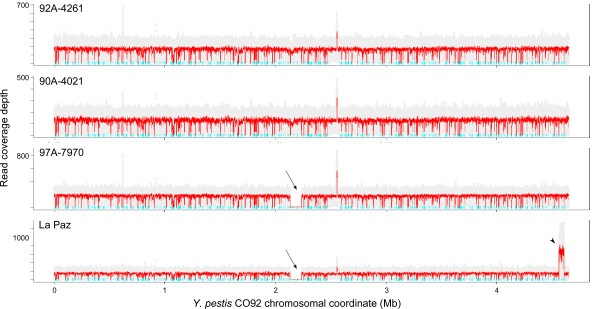
**Coverage plots of *Y. pestis *chromosomes**. Reads were mapped to the *Y. pestis *CO92 chromosome using mapreads. Each row represents a *Y. pestis *chromosome (strain indicated in the upper left of each plot) as described in Figure 1. The arrow in the 97A-7970 and La Paz plots indicates the deletion region depicted in detail in Figure 7. The arrowhead in the La Paz plot indicates the amplified region depicted in Figure 8.

A similar resequencing approach was taken for discovery of polymorphisms in *Y. pestis *strains. Fragment libraries of four *Y. pestis *strains (three North American and one Bolivian strain, all from the 1.ORI lineage) were sequenced, each on one-eighth of a SOLiD™ system flow cell, together with four *B. anthracis *strains (see above). *Y. pestis *reads were mapped against the published reference genome (chromosome and three plasmids) of another *Y. pestis *North American Orientalis strain, CO92. Between 25 million and 36 million reads of 35 bp were mapped for each strain, with median chromosomal read depth ranging from 129 for 90A-4021 to 176 for 97A-7970 (Table 4, Figure 5). Median coverage of the plasmids pMT1 and pCD1 suggested mean copy numbers of 1 to 2 per chromosome, whereas the copy number of the pPCP1 plasmid ranged from 10 to 17 per chromosome. Fewer than 320,000 reference bases were not covered by a uniquely matching read, with most of these bases being found in repetitive or multi-copy genomic sequence (for example, rRNA loci and high copy number insertion elements).

**Table 4:** *Y. pestis *SOLiD™ system sequencing statistics

	92A-4261 (CA)	90A-4021 (CA)	97A-7970 (CA)	La Paz (Bolivia)
Beads found, n	34,339,098	25,716,321	36,469,495	28,150,279

Uniquely placed beads (≤ 3 mismatches), n (%)	22,951,932 (66.84)	17,593,054 (68.41)	23,353,330 (64.04)	19,273,600 (68.47)

Bases not uniquely covered, n (%)	242925 (5.03)	243881 (5.05)	243543 (5.04)	316674 (6.56)

Chromosome median coverage*^a^*	168×	129×	176×	139×

pCD1 median coverage	379×	292×	328×	141×

pCD1 mean copy number, n	2.3	2.3	1.9	1.0

pPCP1 median coverage	2,350×	1,283×	1,694×	2,339×

pPCP1 mean copy number	14.0	9.9	9.6	16.8

pMT1 median coverage	241×	185×	248×	119×

pMT1 mean copy number	1.4	1.4	1.4	0.9

^a^6.7% of the reference genome is non-unique 35-mers.

×, times.

**Figure 6:**
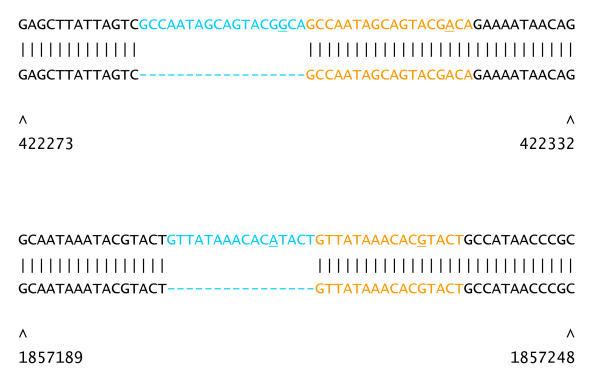
**Two false-positive SNPs in *Y. pestis *map to imperfect repeat regions**. (Top row) CO92 reference and (bottom row) non-reference genome, with CO92 coordinates indicated below. Repeat units are colored in alternating blue and orange text. (Top panel) An 18 bp tandem repeat in *Y. pestis *La Paz; (bottom panel) 16 bp tandem repeat in *Y. pestis *90A-4021. Bases that differ from the consensus sequence are underlined.

Alignment of reads to the CO92 genome facilitated the detection of SNPs in each of the four *Y. pestis *strains, with between 20 and 54 unambiguous chromosomal SNPs, and at most 9 ambiguities found (Table 5; Additional File 2). Ten putative SNPs found in all four strains were previously found to be errors in the reported reference sequence (DMW and PK, see Additional File 3) and were therefore omitted from the list of SNPs reported here. Up to ten private SNPs were selected from each of the four *Y. pestis *strains for validation by PCR amplification and Sanger sequencing. Of these 22 SNPs, 19 were validated. Two of the SNP calls were found to be associated with the loss of a direct near-perfect repeat copy from a VNTR locus (16 or 18 bp; Figure 6), whereas only one private SNP appeared to be a genuine false-positive call.

**Table 5:** SNPs in *Y. pestis *strains, relative to CO92

	92A-4261	90A-4021	97A-7970	La Paz
Chromosome				
SNPs, n	24	19	28	53
Ambiguities, n	1	1	5	9
Total calls, n	25	20	33	62

pCD1				
SNPs, n	0	0	1	0
Ambiguities, n	0	0	0	0
Total calls, n	0	0	1	0

pPCP1				
SNPs, n	0	0	0	0
Ambiguities, n	0	0	0	1
Total calls, n	0	0	0	1

pMT1				
SNPs, n	1	1	1	1
Ambiguities, n	0	0	0	0
Total calls, n	1	1	1	1

SNP, single-nucleotide polymorphism.

**Figure 7:**
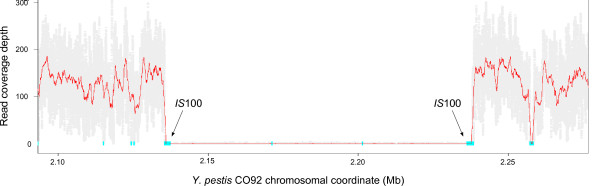
**Common deletion in *Y. pestis *97A-7970 and La Paz**. The 102 kb deletion is depicted on the La Paz chromosome, as in Figure 1. Positions of flanking *IS*100 elements on the CO92 chromosome are indicated.

Alignment of reads to the reference genome identified a shared deletion in 97A-7970 and La Paz, encompassing YPO1902 to YPO1967 of the CO92 genome, which is commonly found in *Y. pestis *strains propagated in the laboratory (Figure 7) [14-17]. PCR across the putative deletion breakpoint followed by Sanger sequencing verified the deletion and confirmed that it is the result of recombination between flanking IS*100 *elements (data not shown).

**Figure 8:**
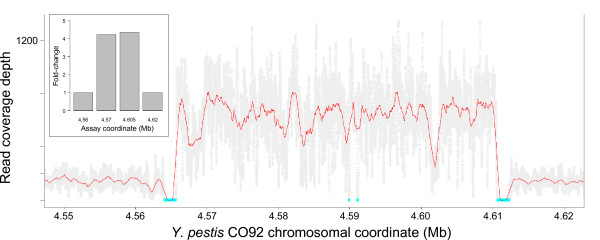
**Validation of amplification in *Y. pestis *strain La Paz**. The amplified region is depicted as in Figure 1. Positions of *IS*1661 elements on the CO92 chromosome are indicated by cyan bars flanking the amplification. Inset: TaqMan^® ^assays were designed, two internal to the amplified region and one to each side, with the approximate coordinates indicated on the X-axis. These assays were run in triplicate on genomic DNA from each of the two strains, and relative fold-change of La Paz versus CO92 was calculated and normalized to the assay at coordinate 4,560,000.

Read coverage depth also revealed an apparent copy number increase in La Paz encompassing CO92 ORFs YPO4048 to YPO4090 (Figure 8). TaqMan^® ^quantitative PCR assays targeting this region indicated that the region is present at a copy number four-fold higher than that of the flanking chromosomal region (Figure 8, inset). Using PCR primers immediately internal to the putative breakpoint and facing out of the region amplified a product containing an IS*1661 *element, suggesting that the amplification is due to direct duplication mediated by recombination between flanking IS*1661 *elements (data not shown). The amplified region of the genome harbors the gene encoding MdfA, a multidrug translocase, suggesting that La Paz (or at least the isolate used in this study) could have increased antibiotic resistance relative to other *Y. pestis *strains.

## Discussion

*B. anthracis *is a clonal pathogen that emerged from a *B. cereus *relative. The genetic differences related to this emergence include at least one important chromosomal mutation (*plcR*) and the acquisition of two virulence plasmids, pXO1 and pXO2, which transformed a soil-living opportunistic pathogen into one capable of catastrophic disease in mammals. The great success of this new pathogen in its new niche has eliminated recombination, as evidenced by whole-genome SNP analysis [12]. Whole-genome SNP discovery was guided by an unbiased genotyping analysis of a large strain collection [10,18] and then followed by extensive SNP genotyping [12,19]. This has established one of the most accurate phylogenetic reconstructions of any bacterial species [18]. SNP genotypes are now the gold standard in *B. anthracis *subtyping, and their power is only limited by the genotyping technology. The future is clearly the performance of whole-genome genotyping with a focus on the SNPs. A whole-genome approach eliminates discovery bias, assays for thousands of known SNPs, and discovers novel SNPs that will be strain-specific [13]. In the case of a bioterrorism event, strain-specific identifiers provide a valuable tool for tracking a biocrime (inclusion) and eliminating (exclusion) natural disease events from the investigative process. As in human forensic investigations, the exclusionary power is tremendously important, and its value in an investigation should not be underestimated.

*Y. pestis *is a very young species that probably arose during the past 20,000 years [20]. As a result, there has been very little time for genetic mutations that can be used as genetic markers to accumulate within this species as a whole. For example, a multilocus sequence typing (MLST) analysis of globally diverse strains found no variation at six loci [21]. This is especially the case for some of the molecular groups within *Y. pestis*. The best example of this is the 1.ORI group, which experienced a global expansion when it was spread from coastal regions in China to Africa, Europe, North America, South America and Australia during the third plague pandemic [22]. Despite its global distribution, the 1.ORI group is nearly monomorphic, because of the historical bottleneck that occurred in China. In addition, specific populations that are the result of this global expansion of the 1.ORI group, such as North American and South American populations, are even more monomorphic, as they may be the result of a single introduction event [23]. Previous whole-genome comparisons using Sanger sequences indicated that phylogenetically informative SNPs can be identified in multiple strains from these very recent populations, such as North American *Y. pestis *[24,25]. Our results demonstrate that MPS can also be used to identify these types of differences but much more rapidly and cheaply than by Sanger sequencing.

We have demonstrated the rapid and accurate simultaneous resequencing of eight bacterial genomes in a single flow cell run of a MPS instrument. The accuracy conferred by two-base encoding and emulsion PCR and the high depth of coverage obtained from the SOLiD™ system allowed most SNPs to be easily distinguished between closely related strains of *B. anthracis *and *Y. pestis*. The results of this study demonstrate the feasibility of using massively parallel sequencing technology to perform whole-genome strain typing to obtain accurate results while scanning the whole-genome of several strains simultaneously. Similar analyses, using Solexa or 454 sequencing, of *Escherichia coli *clones derived from a long-term evolution experiment [26], *Francisella tularensis *clinical isolates [27], or *Salmonella *Typhi clinical isolates [28] further highlight the ability of MPS to identify essentially all genetic differences between closely related strains.

Of the rare SNP calling errors encountered in this study, many are associated with repeat sequences. False-positive SNP calls are often localized to repeat regions or multicopy sequences in the genome. When sequences are identical, unique mapping of reads derived from them is not possible and false-positive SNPs are not called. However, when the sequences differ only by a small number of bases, reads derived from one copy may incorrectly map to a different copy, and this can result in false-positive SNP calls. This phenomenon accounts for the majority of the false-positive SNPs detected in this study. Although these false-positive SNPs are technically errors, they often do lead to the identification of indel polymorphisms (for example, VNTRs) that could be valuable loci for discriminating strains or isolates. Repeats can also impair detection of real SNPs (that is, false-negatives), as was observed in the mapping of A2012 (Ames ancestor) reads to the *B. anthracis *Sterne genome.

Fragment libraries are well suited to the detection of SNPs in very closely related genomes, such as the *B. anthracis *or *Y. pestis *strains analyzed here. They can be constructed from a small amount of genomic DNA and are amenable to barcoding for greater throughput. However, fragment libraries are not as well suited as mate-pair libraries for detection of genomic rearrangements or identification of polymorphisms in non-unique regions of the genome. By allowing otherwise unmappable reads to be unambiguously anchored to a unique region of the genome, mate-pairs facilitate indel detection and result in higher sensitivity for detection of clustered SNPs. Furthermore, long-distance linkage between paired reads allows *de novo *assembly, which may reveal sequences not present in the reference genome, and inference of large chromosomal rearrangements. The use of mate-pair libraries in this study could have eliminated errors caused by incorrect mapping of reads to multiple copies of a nearly identical sequence at different locations in the genome. Mate-pair libraries, however, are unlikely to eliminate errors in tandem repeat sequences unless the repeat units are large and the size of the 'insert' between the two reads is very tightly constrained. Preparation of mate-pair libraries is more complicated and requires more starting material, and barcoding of mate-pair libraries is not currently possible on the SOLiD™ system, so when speed and throughput are important and a very closely related reference genome is available, fragment libraries may be preferable. The choice of whether to use fragment or mate-pair libraries should be guided by the types of polymorphisms to be detected, and the expected degree of relatedness between the strains of interest and their respective reference sequences.

Well-defined reference samples are crucial for comparative genomics studies and assay development, but it is well known that genome sequences deposited in public databases contain errors. For example, in this study, *Y. pestis *reads were mapped against the published genomic sequence of strain CO92. Ten putative SNPs that were common to all four test strains had been previously identified by directed resequencing as errors in the CO92 sequence (DMW and PK, unpublished data, Additional File 3) and therefore could be filtered from the list of polymorphisms. Without this prior knowledge, a researcher would come to the wrong conclusion regarding the degree of variation and what constitutes an informative marker. Given the high throughput and accuracy of the SOLiD™ system, it may be beneficial, particularly for biosafety and biosecurity programs, to re-analyze selected reference genomes, so that high-quality, error-corrected reference standards can be publicly available.

The morphological variants found in the evidence from the 2001 anthrax attacks were present as minority components in the mixture, with much lower prevalence than the wild type, making them potentially difficult to detect. MPS could also be applied to this scenario to rapidly and effectively type these low-level variants and substantially reduce the false-negative rate due to stochastic sampling. Consider the situation where each of the variable loci can be amplified by PCR, yielding an amplicon of approximately 200 bp in length. The combined length for a four-variant positive sample would be 800 bp. Sequencing of these amplicons on the SOLiD™ system would yield a theoretical coverage depth of approximately 12.5 million, assuming throughput of 10 GB per fragment run. By multiplexing amplicons from 256 specimens, coverage would still be approximately 50,000 times, which is estimated to be sufficient for detection of variants present at fewer than 1 in 10,000 in a mixed sample. It may also be possible to identify minority variants, albeit with lower sensitivity, by directly sequencing mixed genomic DNA samples, as recently demonstrated for *E. coli *[29] and *Salmonella *Typhi [30].

In this study, eight genomic DNA samples were analyzed on a single SOLiD™ system flow cell using barcoding to allow multiplexing of samples. Consumables for this experiment had a list price of less than US$8,000, so the reagent cost per genome was less than US$1,000. Using 208 million mapped reads with 35 bp read length, the average coverage obtained in this study was approximately 100-fold. Conservatively assuming that it is possible to get accurate SNP calls with 50-fold coverage ([31], data not shown), the number of strains that can be analyzed in parallel can be doubled to 16. Furthermore, with the current SOLiD™ 3.0 system specifications, it is possible to obtain 300 million mapped reads of 50 bases or approximately twice the coverage obtained here. Thus, it is technically possible to sequence at least 32 strains simultaneously on a single flow cell for approximately US$250 per genome in consumable costs. The efforts required by a skilled team to obtain DNA samples, construct libraries, analyze data and validate results constitute a significant expense that does not scale as readily as instrument reagents, so further multiplexing of samples will require the development of improved automation protocols and analytical tools.

## Conclusions

These results demonstrate that in the event of another bioterrorism case or public health concern, whole-genome sequences of several isolates could be obtained with high accuracy within a matter of days, particularly for viable, culturable specimens. Because of the lower cost, that is, as much as 250 times less than was possible during the initial anthrax investigation, combined with the high coverage (10-100 times greater than shotgun sequencing) and lower error (> 99.9999% accuracy), large repositories could be completely sequenced. With dedicated efficient software for alignment and annotation, and recognition of refractory sequencing results at nominal parts of the genome, sequencing of whole genomes can now be used as a diagnostic and analytical tool for forensic and epidemiologic investigations, rather than just as a research tool.

## Methods

### Bacterial strains

The *B. anthracis *isolates used in this study consisted of four unique strains. The first, A0032, is an isolate from Changping, China, which falls in the A.Br.008/009 can SNP group [19]. The second, A0324, is a strain originating from Slovakia, and is also of the A.Br.008/009 can SNP group, which was described by Okinaka *et al. *[32] as having a unique plasmid SNP in common with only a few other strains from this particular region. The next isolate, A0377, originates from Haiti and is consistent with the A.Br.WNA lineage; it falls within the large (236 isolates) subclade node that is located third from the terminal node, based on the sequenced strain described by Kenefic *et al. *[33]. The final strain, A2012, was isolated from the first fatality victim, Robert Stevens, during the 2001 letter attacks. This is the Ames strain that was used in these attacks and was identified as this strain via multilocus VNTR analysis 8 genotyping [10].

Four *Yersinia pestis *strains were examined in this study: 92A-4261, 90A-4021, 97A-7970 and La Paz. Strain 92A-4261 was isolated from a flea pool collected in Plumas County, California in August 1992. Strain 90A-4021 was isolated in Siskiyou County, California in July 1990 from a chipmunk. Strain 97A-7070 was isolated from a flea pool collected in San Bernardino County, California in July 1997. The La Paz strain was isolated from a human in 1969 in La Paz, Bolivia. All four of these strains are assigned to the 1.ORI group, which spread around the world during the third plague pandemic in the 1800 s and 1900 s [20].

### Genomic DNA preparation

Genomic DNA was prepared as follows (all DNA isolation reagents were obtained from Sigma Chemical Co., St. Louis, MO, USA unless otherwise stated). Lawn cultures from single colonies of each isolate were prepared by streaking onto plates of Trypticase Soy AGar with 5% sheep blood (Hardy Diagnostics, Santa Maria, CA, USA) followed by 20 hours of incubation at 37°C for *B. anthracis *or 48 hours of incubation at 28°C for *Y. pestis*. Cells were harvested and suspended in 11 ml of TE buffer (10 mM Tris pH 8.0, 1 mM EDTA). The cell suspensions were frozen in liquid nitrogen for ~2 min and then thawed in a water bath at 65°C. This freeze-thaw step was repeated twice, giving for a total of three cycles. To each extraction lysate, 225 μl of 20% sodium dodecyl sulfate and 45 μl of proteinase K 20 mg/ml were added, and mixed gently for 5 min. This step was followed by a 2 hour incubation at 55°C. Next, 2.5 ml of 5 M NaCl were added and the mixture was mixed by rocking for 5 min. To the suspension, 1.4 ml of 10% (w/v) hexadecyltrimethylammonium bromide (CTAB) in 0.7 M NaCl were added, and mixed thoroughly by inversion followed by a 10 minute incubation at 65°C. After incubation, 12 ml chloroform-isoamyl alcohol (24:1) were added, and the solution gently mixed by inversion for 10 min followed by a centrifugation step for 10 minutes at 4°C at 3,220 *g*. The upper aqueous phase was then collected and again extracted with chloroform:isoamyl alcohol. After the final centrifugation step, the upper aqueous phase was collected and isopropanol (0.6 v/v) was added for DNA precipitation overnight at -20°C. The precipitated DNA was then pelleted at 4°C and 3,220 g for 30 minutes. The supernatant was removed, and 1 ml of 70% ethanol was added to wash the DNA pellet. The DNA was then re-pelleted at 4°C at 3,220 g for 10 minutes, the was supernatant removed, and the pellet briefly dried and resuspended in 500 μl of TE buffer.

Genomic DNA extracts were quantified using a commercial kit for double stranded DNA (PicoGreen^®^; Molecular Probes, Inc., Eugene, OR, USA) according to the manufacturer's recommendations. Sample fluorescence was then measured using a fluorometer (Gemini XPS; Molecular Devices, Inc., Sunnyvale, CA, USA).

### SOLiD™ system sequencing

Barcoded fragment libraries were constructed for each of the *B. anthracis *and *Y. pestis *strains as follows. Genomic DNA (1.5 to 2 μg) was sheared, end-repaired (End-It; Epicentre Biotechnologies, Madison, WI, USA) and ligated to barcoded sequencing adapters. Construct fragments of 175 to 200 bp in size were selected and purified from a 6% PAGE gel. Resulting libraries were nick-translated and amplified using 11 to 17 cycles of PCR, before purification and quantification (2100 Bioanalyzer; Agilent Technologies, Santa Clara, CA, USA). Libraries were pooled in equimolar ratios, and templated beads were generated by emulsion PCR before deposition onto a single SOLiD™ system flow cell. Sequencing was carried out to 35 base pairs using SOLiD™ 3 chemistry (Applied Biosystems, Foster City, CA, USA).

### Data analysis

Alignment of SOLiD™ system reads to *B. anthracis *Ames ancestor (Refseq accessions NC_007530.2, NC_007322.2 and NC_007323.3), *B. anthracis *Sterne (Refseq accession NC_005945.1) or *Y. pestis *CO92 (Refseq accessions NC_003143.1, NC_003131.1, NC_003132.1 and NC_003134.1), was performed using the SOLiD™ System Analysis Pipeline Tool http://solidsoftwaretools.com/gf/project/corona/, allowing a maximum of three colorspace mismatches per read (the core alignment program within this pipeline is mapreads). SNPs were called using either diBayes http://solidsoftwaretools.com/gf/project/dibayes/ or the SOLiD™ System Analysis Pipeline Tool. Heterozygous SNPs reported by the software were interpreted as ambiguous, as bacteria are haploid. SNP identification between the *B. anthracis *Ames ancestor and Sterne finished chromosome sequences was performed using the MUMmer suite [34]. The genome alignment in the GBAA_1572 region of *B. anthracis *was manually edited to obtain the proper alignment of 27 bp repeat units (see Figure 2B). Further investigation of putative false-positive SNPs also led to the confirmation of four SNPs that were not called by MUMmer because of their localization within repeat sequences.

### Sanger sequencing

Up to 10 private SNPs were selected from each of the three previously unsequenced strains. Flanking PCR primers were designed 250 to 300 nucleotides to either side of each SNP and used for PCR amplification followed by bidirectional Sanger sequencing (BigDye^® ^Terminator v1.1 Cycle Sequencing Kit) on a DNA analyzer (3730x1 Applied Biosystems, Foster City, CA, USA). SNPs were validated by the alignment of Sanger contigs to the Ames ancestor genomic sequence and SNP detection using MUMmer [34].

### Quantitative real-time PCR

Four TaqMan^® ^assays were designed for each putative copy number variation locus: two within the amplified region and one flanking each end of the amplified region. MGB probes were labeled with 6-FAM (see Additional File 4). Real-time amplification was performed using TaqMan^® ^Universal PCR Master Mix and a real-time PCR system (Model 7500; Applied Biosystems). Reactions were run in triplicate on strains with and without the copy number variation, and fold-change values were calculated using the ΔΔC_t _method [35].

## Competing interests

CAC, CABC, RF, MB, PB and MRF are employees of Life Technologies Corporation, maker of the Applied Biosystems SOLiD™ system. No other competing interests are declared.

## Authors' contributions

PK, BB, MF and CAC conceived the experiments. CABC and MB prepared libraries and performed SOLiD™ system sequencing. CAC coordinated the experimental work and analyzed the data. RF performed directed sequencing and real-time PCR validation experiments. MM, JB, JS, DB and AV selected strains, cultured bacteria and prepared genomic DNA. PB participated in data analysis. CAC, PK, BB, PW and DW drafted the manuscript. All authors read and approved the final manuscript.

## Supplementary Material

Additional file 1 ***B. anthracis *SNPs**. A table in Microsoft Excel format of *B. anthracis *SNPs identified in this study by SOLiD™ system sequencing. Click here for file

Additional file 2 ***Y. pestis *SNPs**. A table in Microsoft Excel format of *Y. pestis *SNPs identified in this study by SOLiD™ system sequencing. Click here for file

Additional file 3 ***Y. pestis *CO92 sequence corrections**. A table in PDF format of corrections made to the *Y. pestis *CO92 reference chromosome sequence. Click here for file

Additional file 4 **TaqMan^® ^oligonucleotide sequences**. A table in PDF format of assay oligonucleotide sequences used for real-time PCR verification of putative genomic amplification regions. Click here for file

## References

[B1] Budowle B Schutzer SE Ascher MS Atlas RM Burans JP Chakraborty R Dunn JJ Fraser CM Franz DR Leighton TJ Morse SA Murch RS Ravel J Rock DL Slezak TR Velsko SP Walsh AC Walters RA Toward a system of microbial forensics: from sample collection to interpretation of evidence Appl Environ Microbiol 2005 71 2209 2213 10.1128/AEM.71.5.2209-2213.2005 15870301 PMC1087589

[B2] Cummings CA Relman DA Genomics and microbiology. Microbial forensics--"cross-examining pathogens" Science 2002 296 1976 1979 10.1126/science.1073125 12004075

[B3] Amerithrax Investigation Court Documents http://www.fbi.gov/page2/august08/amerithrax_docs080608.html

[B4] Ravel J Jiang L Stanley ST Wilson MR Decker RS Read TD Worsham P Keim PS Salzberg SL Fraser-Liggett CM Rasko D The complete genome sequence of *Bacillus anthracis *Ames "Ancestor" J Bacteriol 2009 191 445 446 10.1128/JB.01347-08 18952800 PMC2612425

[B5] Read TD Peterson SN Tourasse N Baillie LW Paulsen IT Nelson KE Tettelin H Fouts DE Eisen JA Gill SR The genome sequence of *Bacillus anthracis *Ames and comparison to closely related bacteria Nature 2003 423 81 86 10.1038/nature01586 12721629

[B6] Read TD Salzberg SL Pop M Shumway M Umayam L Jiang L Holtzapple E Busch JD Smith KL Schupp JM Solomon D Keim P Fraser CM Comparative genome sequencing for discovery of novel polymorphisms in *Bacillus anthracis* Science 2002 296 2028 2033 10.1126/science.1071837 12004073

[B7] Schutzer SE Keim P Czerwinski K Budowle B Use of forensic methods under exigent circumstances without full validation Sci Transl Med 2009 1 8cm7 20368183 10.1126/scitranslmed.3000372

[B8] MacLean D Jones JD Studholme DJ Application of 'next-generation' sequencing technologies to microbial genetics Nat Rev Microbiol 2009 7 287 296 19287448 10.1038/nrmicro2122

[B9] Mardis ER The impact of next-generation sequencing technology on genetics Trends Genet 2008 24 133 141 18262675 10.1016/j.tig.2007.12.007 PMC2680276

[B10] Keim P Price LB Klevytska AM Smith KL Schupp JM Okinaka R Jackson PJ Hugh-Jones ME Multiple-locus variable-number tandem repeat analysis reveals genetic relationships within *Bacillus anthracis* J Bacteriol 2000 182 2928 2936 10.1128/JB.182.10.2928-2936.2000 10781564 PMC102004

[B11] Keim P Van Ert MN Pearson T Vogler AJ Huynh LY Wagner DM Anthrax molecular epidemiology and forensics: using the appropriate marker for different evolutionary scales Infect Genet Evol 2004 4 205 213 10.1016/j.meegid.2004.02.005 15450200

[B12] Pearson T Busch JD Ravel J Read TD Rhoton SD U'Ren JM Simonson TS Kachur SM Leadem RR Cardon ML Van Ert MN Huynh LY Fraser CM Keim P Phylogenetic discovery bias in *Bacillus anthracis *using single-nucleotide polymorphisms from whole-genome sequencing Proc Natl Acad Sci USA 2004 101 13536 13541 10.1073/pnas.0403844101 15347815 PMC518758

[B13] Van Ert MN Easterday WR Huynh LY Okinaka RT Hugh-Jones ME Ravel J Zanecki SR Pearson T Simonson TS U'Ren JM Kachur SM Leadem-Dougherty RR Rhoton SD Zinser G Farlow J Coker PR Smith KL Wang B Kenefic LJ Fraser-Liggett CM Wagner DM Keim P Global genetic population structure of *Bacillus anthracis* PLoS One 2007 2 e461 10.1371/journal.pone.0000461 17520020 PMC1866244

[B14] Hinchliffe SJ Isherwood KE Stabler RA Prentice MB Rakin A Nichols RA Oyston PC Hinds J Titball RW Wren BW Application of DNA microarrays to study the evolutionary genomics of *Yersinia pestis *and *Yersinia pseudotuberculosi*s Genome Res 2003 13 2018 2029 10.1101/gr.1507303 12952873 PMC403674

[B15] Fetherston JD Schuetze P Perry RD Loss of the pigmentation phenotype in *Yersinia pestis *is due to the spontaneous deletion of 102 kb of chromosomal DNA which is flanked by a repetitive element Mol Microbiol 1992 6 2693 2704 10.1111/j.1365-2958.1992.tb01446.x 1447977

[B16] Fetherston JD Perry RD The pigmentation locus of *Yersinia pestis *KIM6+ is flanked by an insertion sequence and includes the structural genes for pesticin sensitivity and HMWP2 Mol Microbiol 1994 13 697 708 10.1111/j.1365-2958.1994.tb00463.x 7997181

[B17] Buchrieser C Prentice M Carniel E The 102-kilobase unstable region of *Yersinia pestis *comprises a high-pathogenicity island linked to a pigmentation segment which undergoes internal rearrangement J Bacteriol 1998 180 2321 2329 9573181 10.1128/jb.180.9.2321-2329.1998 PMC107171

[B18] Pearson T Okinaka RT Foster JT Keim P Phylogenetic understanding of clonal populations in an era of whole genome sequencing Infect Genet Evol 2009 9 1010 9 10.1016/j.meegid.2009.05.014 19477301

[B19] Van Ert MN Easterday WR Simonson TS U'Ren JM Pearson T Kenefic LJ Busch JD Huynh LY Dukerich M Trim CB Beaudry J Welty-Bernard A Read T Fraser CM Ravel J Keim P Strain-specific single-nucleotide polymorphism assays for the *Bacillus anthracis *Ames strain J Clin Microbiol 2007 45 47 53 10.1128/JCM.01233-06 17093023 PMC1828967

[B20] Achtman M Carniel E, Hinnebusch BJ Age, descent and genetic diversity within *Yersinia pestis* Yersinia: Molecular and Cellular Biology 2004 Norwich, UK: Horizon Bioscience 432

[B21] Achtman M Zurth K Morelli G Torrea G Guiyoule A Carniel E *Yersinia pestis*, the cause of plague, is a recently emerged clone of *Yersinia pseudotuberculosis* Proc Natl Acad Sci USA 1999 96 14043 14048 10.1073/pnas.96.24.14043 10570195 PMC24187

[B22] Keim PS Wagner DM Humans and evolutionary and ecological forces shaped the phylogeography of recently emerged diseases Nat Rev Microbiol 2009 7 813 821 10.1038/nrmicro2219 19820723 PMC2794044

[B23] Vogler AJ Driebe EM Lee J Auerbach RK Allender CJ Stanley M Kubota K Andersen GL Radnedge L Worsham PL Keim P Wagner DM Assays for the rapid and specific identification of North American *Yersinia pestis *and the common laboratory strain CO92 Biotechniques 2008 44 201, 203-204, 207 10.2144/000112815 18330347 PMC3836605

[B24] Auerbach RK Tuanyok A Probert WS Kenefic L Vogler AJ Bruce DC Munk C Brettin TS Eppinger M Ravel J Wagner DM Keim P *Yersinia pestis *evolution on a small timescale: comparison of whole genome sequences from North America PLoS One 2007 2 e770 10.1371/journal.pone.0000770 17712418 PMC1940323

[B25] Touchman JW Wagner DM Hao J Mastrian SD Shah MK Vogler AJ Allender CJ Clark EA Benitez DS Youngkin DJ Girard JM Auerbach RK Beckstrom-Sternberg SM Keim P A North American *Yersinia pestis *draft genome sequence: SNPs and phylogenetic analysis PLoS One 2007 2 e220 10.1371/journal.pone.0000220 17311096 PMC1794153

[B26] Barrick JE Yu DS Yoon SH Jeong H Oh TK Schneider D Lenski RE Kim JF Genome evolution and adaptation in a long-term experiment with *Escherichia coli* Nature 2009 461 1243 1247 10.1038/nature08480 19838166

[B27] La Scola B Elkarkouri K Li W Wahab T Fournous G Rolain JM Biswas S Drancourt M Robert C Audic S Löfdahl S Raoult D Rapid comparative genomic analysis for clinical microbiology: the *Francisella tularensis *paradigm Genome Res 2008 18 742 750 10.1101/gr.071266.107 18407970 PMC2336804

[B28] Holt KE Parkhill J Mazzoni CJ Roumagnac P Weill FX Goodhead I Rance R Baker S Maskell DJ Wain J Dolecek C Achtman M Dougan G High-throughput sequencing provides insights into genome variation and evolution in *Salmonella Typhi* Nat Genet 2008 40 987 993 10.1038/ng.195 18660809 PMC2652037

[B29] Barrick JE Lenski RE Genome-wide Mutational Diversity in an Evolving Population of *Escherichia coli* Cold Spring Harb Symp Quant Biol 2009 in press 19776167 10.1101/sqb.2009.74.018 PMC2890043

[B30] Holt KE Teo YY Li H Nair S Dougan G Wain J Parkhill J Detecting SNPs and estimating allele frequencies in clonal bacterial populations by sequencing pooled DNA Bioinformatics 2009 25 2074 2075 10.1093/bioinformatics/btp344 19497932 PMC2722999

[B31] Smith DR Quinlan AR Peckham HE Makowsky K Tao W Woolf B Shen L Donahue WF Tusneem N Stromberg MP Stewart DA Zhang L Ranade SS Warner JB Lee CC Coleman BE Zhang Z McLaughlin SF Malek JA Sorenson JM Blanchard AP Chapman J Hillman D Chen F Rokhsar DS McKernan KJ Jeffries TW Marth GT Richardson PM Rapid whole-genome mutational profiling using next-generation sequencing technologies Genome Res 2008 18 1638 1642 10.1101/gr.077776.108 18775913 PMC2556265

[B32] Okinaka RT Henrie M Hill KK Lowery KS Van Ert M Pearson T Schupp J Kenefic L Beaudry J Hofstadler SA Jackson PJ Keim P Single nucleotide polymorphism typing of *Bacillus anthracis *from Sverdlovsk tissue Emerg Infect Dis 2008 14 653 656 10.3201/eid1404.070984 18394287 PMC2570946

[B33] Kenefic LJ Beaudry J Trim C Daly R Parmar R Zanecki S Huynh L Van Ert MN Wagner DM Graham T Keim P High resolution genotyping of *Bacillus anthracis *outbreak strains using four highly mutable single nucleotide repeat markers Lett Appl Microbiol 2008 46 600 603 10.1111/j.1472-765X.2008.02353.x 18363651

[B34] Kurtz S Phillippy A Delcher AL Smoot M Shumway M Antonescu C Salzberg SL Versatile and open software for comparing large genomes Genome Biol 2004 5 R12 10.1186/gb-2004-5-2-r12 14759262 PMC395750

[B35] Livak KJ Schmittgen TD Analysis of relative gene expression data using real-time quantitative PCR and the 2^ΔΔCt ^Method Methods 2001 25 402 408 10.1006/meth.2001.1262 11846609

