# Supplementary material for: Accurate, rapid and high-throughput detection of strain-specific polymorphisms in Bacillus anthracis and Yersinia pestis by next-generation sequencing
Source: Investig Genet. 2010 Sep 1;1:5. doi: 10.1186/2041-2223-1-5 (PMC2988479; doi:10.1186/2041-2223-1-5)
Supplement: Additional file 3 — Y. pestis CO92 sequence corrections. A table in PDF format of corrections made to the Y. pestis CO92 reference chromosome sequence. [file 2041-2223-1-5-S3.PDF]

Additional File 3: *Y. pestis* CO92 sequence corrections

CO92 coordinate	Gene	CO92 Refseq NC_003143.1 (error)	CO92 Keim Lab (corrected)	SOLiD™ system consensus
150,946	YPO0138	C	A	A
351,821	YPO0342	T	G	G
917,155	YPO0837	A	G	G
1,939,828	YPO1701	T	G	G
1,939,841	YPO1701	A	G	G
2,273,616	YPO2000	G	C	C
3,608,932	YPO3243	T	C	C
3,647,867	YPO3273	C	T	T
3,655,609	YPO3275	T	C	C
4,624,135	YPO4103	C	G	G
