# Supplementary material for: Accurate, rapid and high-throughput detection of strain-specific polymorphisms in Bacillus anthracis and Yersinia pestis by next-generation sequencing
Source: Investig Genet. 2010 Sep 1;1:5. doi: 10.1186/2041-2223-1-5 (PMC2988479; doi:10.1186/2041-2223-1-5)
Supplement: Additional file 4 — TaqMan® oligonucleotide sequences. A table in PDF format of assay oligonucleotide sequences used for real-time PCR verification of putative genomic amplification regions. [file 2041-2223-1-5-S4.PDF]

Additional File 4: TaqMan oligonucleotide sequences

Assay ID	Forward primer	Reverse primer	MGB Probe
Ba_50000	GGGATTATTGTAAATAATATGTTTTACAGTTCAGGACA	GTTACGTCTCCTGCTACAAGCT	CCACTCGCAGTTAACG
Ba_120000	TGTTGAGCGCGGTATCGTT	CATTTTCTTCAGCAAGACCGATGAT	CTACTACGTCACCAACTTT
Ba_130000	GCGCACAAGTTCTCTTCAAGTG	GTACGAAACATTAGATCACCTCAACTGA	CTGCTTCAATTGCTTCTTTAG
Ba_200000	CCGACACGTGGTAATGAAAGAAGTA	CAAATATGCAATCCGTAACGCTTTATCT	CAGTTCCCCATAGTAAACC
Yp_4560000	ATGCGCCCCACGAGAAT	GCCCGATGCTGGGTAATTCA	TCGCCACTAAAGAATG
Yp_4570000	TGAGGATGTGATAGCGCAATGTC	CAATGAGATGGCGGCATTACTG	CCGGCGACGACAGTATC
Yp_4605000	GCCATGGCGTAACCCATC	GGCTCAGCAAATTCTTACCGATT	CCCATTTGACTGACCAGTTG
Yp_4620000	TCGGAATATACTGGTGTCCCCTT	CGGTGAGATCCTTTGCCGATTAAAA	CCCCTTTCTCAGGCAATG

Assays beginning with Ba_ target the B. anthracis A0377 amplification
Assays beginning with Yp_ target the Y. pestis La Paz amplification
Numbers in assay IDs are approximate chromosomal coordinates
All probes are labeled with 6-FAM at the 5' end and MGB at the 3' end
